# Standardizing Primary Health Care Referral Data Sets in Nigeria: Practitioners' Survey, Form Reviews, and Profiling of Fast Healthcare Interoperability Resources (FHIR)

**DOI:** 10.2196/28510

**Published:** 2022-07-07

**Authors:** Emeka Chukwu, Lalit Garg, Nkiruka Obande-Ogbuinya, Vijay Kumar Chattu

**Affiliations:** 1 Department of Computer Information Systems Faculty of Information Communication Technology University of Malta Msida Malta; 2 Digital Health Interoperability Network Abuja Nigeria; 3 Department of Science Education Alex-Ekwueme Federal University Ndufu-Alike Ikwo Ebonyi Nigeria; 4 Department of Occupational Science & Occupational Therapy Temerty Faculty of Medicine University of Toronto Toronto, ON Canada; 5 Center for Transdisciplinary Research Saveetha Institute of Medical and Technological Sciences Saveetha University Chennai India; 6 Department of Community Medicine Faculty of Medicine Datta Meghe Institute of Medical Sciences Wardha India

**Keywords:** FHIR, COVID-19, digital health, eHealth, mHealth, BlockMom, Nigeria, primary health care, health information, health information exchange, interoperability

## Abstract

**Background:**

Referral linkages are crucial for efficient functioning of primary health care (PHC) systems. Fast Healthcare Interoperability Resource (FHIR) is an open global standard that facilitates structuring of health information for coordinated exchange among stakeholders.

**Objective:**

The objective of this study is to design FHIR profiles and present methodology and the profiled FHIR resource for Maternal and Child Health referral use cases in Ebonyi state, Nigeria—a typical low- and middle-income country (LMIC) setting.

**Methods:**

Practicing doctors, midwives, and nurses were purposefully sampled and surveyed. Different referral forms were reviewed. The union of data sets from surveys and forms was aggregated and mapped to base patient FHIR resource elements, and extensions were created for data sets not in the core FHIR specification. This study also introduced FHIR and its relation to the World Health Organization’s (WHO’s) International Classification of Diseases.

**Results:**

We found many different data elements from the referral forms and survey responses even in urban settings. The resulting FHIR standard profile is published on GitHub for adaptation or adoption as necessary to aid alignment with WHO recommendations. Understanding data sets used in health care and clinical practice for information sharing is crucial in properly standardizing information sharing, particularly during the management of COVID-19 and other infectious diseases. Development organizations and governments can use this methodology and profile to fast-track FHIR standards adoption for paper and electronic information sharing at PHC systems in LMICs.

**Conclusions:**

We presented our methodology for profiling the referral resource crucial for the standardized exchange of new and expectant moms’ information. Using data from frontline providers and mapping to the FHIR profile helped contextualize the standardized profile.

## Introduction

### Background

Health care is a complex sector that involves medical professionals, allied health workers, the information and communication technology (ICT) workforce, and various other stakeholders. The World Health Organization (WHO) highlights the importance of 6 building blocks of any health system: service delivery, health workforce, health information systems, access to essential medicines, financing, and leadership or governance [1]. Therefore, the health information systems block is very critical and plays an important role in data capture, processing, and usage. Substantial investments have been made in the development and strengthening of routine health information systems (RHIS) in many low- and middle-income countries (LMICs) over the past 2 decades [2,3]. Although early RHISs were produced using paper-based health facility reports, many LMICs have implemented newer web-based systems over the past decade [4,5]. Given that substantial investments have been made in strengthening RHISs in LMICs in recent years, researchers have a growing demand for more real-time data [6]. Besides, data for policy and operational decision-making in LMICs, including Nigeria, have been largely limited to report generation, process monitoring, and early surveillance responses. Reliable, quality, timely, and transparent health service data are essential for an efficient health system [7]. Globally, health care interoperability has been identified as vital to seamless care coordination among the different stakeholders.

### Global Health Care Interoperability

According to the World Health Organization (WHO) Europe’s 2016 e-Health in practice report, Estonia is the first country to implement electronic health records (EHRs) [8]. The famous X-Road facilitates Estonia's exchange network, an interoperability layer launched in 2001, with several different services added over the years. Estonia achieved success with over 99% of electronic medical subscriptions in 2018. Estonia's X-Road interoperability layer connects over 2700 services across 700 institutions and enterprises across several sectors, including health care. The United Kingdom’s national service specification was based on the Health Level 7 (HL7) version 3 standard and is now transitioning to the HL7 Fast Healthcare Interoperability Resource (FHIR). However, local implementation was left to providers to determine, most of whom already run different versions of HL7 version 2 [9]. This National Health Service project started with 2 main use cases: the Summary Care Records and the Detailed Care Records [9]. Canada launched a national Infoway project to standardize and foster collaboration among pan-Canadian health care solutions [10]. After leveraging the CEN TC251 standards for referrals, discharge letters, laboratory, prescriptions, reimbursements, radiology requests, and reports, a national program was deemed successful in Denmark. These use cases were pilot-tested via 15 independently managed projects [10]. In 2008, a report highlighted that in the United States, “only 15 to 20 percent of medical doctors have access to computerized patient records and only a small fraction of the billions of medical transactions happen electronically” [10]. Such a low usage led to the creation of Health Information Technology for Economic and Clinical Health, which was launched in 2009 to incentivize digitization, and things have since changed.

### Interoperability in LMICs

Some LMIC health systems services are still paper-dependent for recording and transmitting health information. Paper records are limited because only one person can access them at a time. Systematic digitization of health systems has driven the development and implementation of national digital health strategies in Nigeria and other LMICs [11,12]. From our literature search [13] and to our knowledge, LMICs still struggle with patient-level interoperability project implementations, which has limited recording successes. Nigeria is a typical LMIC because it has one of the highest global burdens of maternal mortality [14]. Furthermore, there are more primary health care (PHC) facilities (approximately 10 times) than hospitals in Nigeria; hence, here we focus on PHCs. PHCs have the highest potential for impact in the Nigerian health system because most health services are delivered at the PHC level. In a typical PHC network, the possible use cases for health information interchange may include the following:

- Interdepartmental care communication
- Inter-PHC or PHC to secondary hospital referral
- Reporting of decentralized laboratory results
- Triangulation of immunization and surveillance information
- Payment settlement
- Diagnostic information exchange

Nigeria used the DHIS2 for routine reporting of the delivery of health information system services. Routine health information systems (RHISs) continue to collect data on a wide range of diseases and conditions [6]. These RHIS data are analyzed to assess community-level initiatives such as policies to boost community engagement and strengthen referrals from traditional birth attendants to increase demand for maternal and child care [15-17]. The COVID-19 pandemic has further exposed the weakness in health systems worldwide and the value of linkages.

### Health Care Interoperability Standards

The international organizations for certifying and ratifying widely used digital health standards are the ISO/TC (International Organization for Standards’ Health Informatics Technical Committee) 215 and CEN/TC (European Committee for Standards’ Health ICT Technical committee) 251. For instance, ISO 21090:2011 is a ratified HL7 version 3 data type for information interchange. Similarly, ISO 13606-1:2019 is a ratified description of archetype reference models. HL7 is a leading health care standard development organization that has facilitated many standards, including the HL7 version 2 messaging standard, HL7 version 3 Clinical Document Architecture document exchange standard, and the HL7 FHIR. FHIR was popularized because it supports REpresentational State Transfer (REST)–based web-based (real-time) transactions and its extension for services. FHIR is now emerging as the de facto global standard for health care data interchange. The FHIR community includes Microsoft, Google, Apple, and many electronic medical record and EHR vendors [18-20]. In addition, the WHO has recently published a digital adaptation kit to support countries deploying standards for antenatal care [21].

### Terminologies

In addition to data interchange standards, terminology categorization helps guarantee consistent and uniform understanding (and meaning) of terms in health care systems (within and across geographies). The leading terminologies for disease, procedure, and other concept classification are Systematized Nomenclature of Medicine–Clinical Terms (SNOMED-CT) and the International Classification of Diseases (ICD). Other technology providers are Logical Observation Identifiers Names and Codes (LOINC) for laboratory result reporting and Digital Imaging and Communication in Medicine (DICOM) for imaging data reporting. This study used ICD because it uses a free license compared to the better developed SNOMED-CT for disease classification. Code systems such as the WHO ICD-10 use statistical classification of medical concepts and entities into coded groups, assigning identifiers [22]. Codes allow for the unique identification of these concepts in an information processing system. These codes classify diseases, procedures, billing, history or symptoms, and case summaries (jurisdictional and international aggregate reporting). Simultaneously, service providers, including clinicians, use clinical terms in information processing tools.

### Study Objectives

The project's main objective is to use a referral use case to profile, validate, and present data elements relevant to exchanging health information at the PHC level of care. Profiling is the strategy for defining FHIR models by domesticating the international core standard through specific use cases by structured authoring and publishing. Global best practices facilitate digital health information exchange for better care by using standardized data. Digital tools can only communicate using data in certain formats (eg, XML or JSON), organized in an agreed structure [23].

## Methods

### Overview

We reviewed paper referral forms and surveyed frontline health workers, drawing inspiration from similar work conducted by Odisho et al [24]. We checked how consistent the referral data sets were. Aggregated referral data sets were then mapped to and FHIR extensions profiled. We also modeled data types and cardinalities, including references to other profiles, resources, and terminology binding to ICD-10.

### Stakeholder Interviews and Data Set Identification

We established the research focus by addressing the data flow in the maternal and child health information flow value chain in Ebonyi State, Nigeria. Nigeria has between 28,000 and 36,000 health facilities overall. Ebonyi state is one of the 36 subregional governments in Nigeria with 171 “functional” PHC centers and 13 general hospitals [25]. Although from the National Health workforce Registry, there are up to 830 health facilities in the state [26]. Based on our use case, a strategic point of data exchange among multiple PHC centers or PHC centers and hospitals is the referral chain for pregnant women. We used the purposeful snowball sampling technique to identify health care providers in Ebonyi State and share the survey questionnaire.

We sent out questionnaires and a request for a copy of “referral forms” was used for 24 health workers in their respective health facilities in Ebonyi State. Between June 10 and 17, 2019, all 24 health workers completed and returned the questionnaires, and only 3 provided referral paper forms. Respondents were a mix of medical doctors, midwives, and nurses, as shown in Table 1.

We acknowledge the possibility of selection bias, and, for instance, these providers were mostly from health facilities in Abakaliki, the state capital and the main metropolitan city. We consider this bias insignificant as we measured consistency or variation in referral data sets among providers, which was significant. Each provider was from a different health facility (except the tertiary hospital doctors).

The structured questionnaire used asked the following questions:

1. What information is shared when referring-out a pregnant woman?
2. What information is expected when referring-in a pregnant woman?
3. What forms are used, and what are the contents of these forms?
4. What information is the client or caregiver expected to know or have at the recipient end?

**Table 1:** Distribution of respondents and their roles.

Workstation	Roles, n		
	Nurse	Midwife	Doctor
Primary health care clinics	3	3	4
Secondary health care (general hospitals)	4	2	3
Tertiary health care (teaching hospital)	0	1	3
State Ministry of Health	0	1	0

### Profiling, Validation, and Publishing

**Figure 1:**
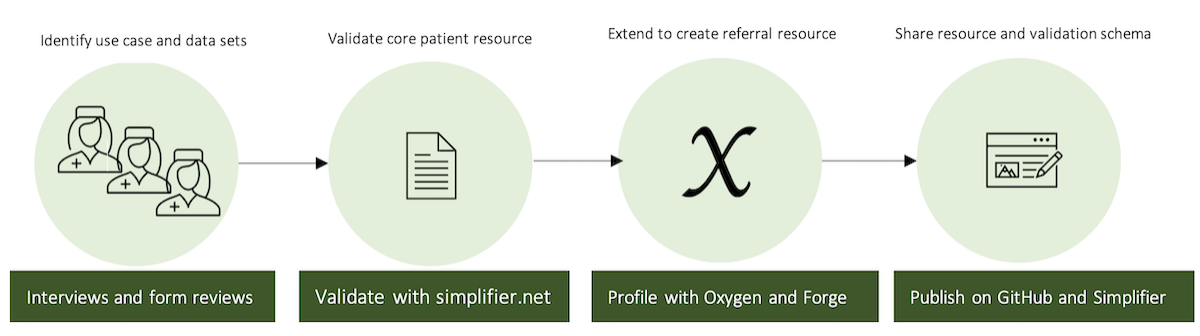
Steps to profiling and publishing the Fast Healthcare Interoperability Resource.

We started by creating a default patient profile with no extension by using the Forge tool and uploading it on the simplifier.net web interface under the BlockMom project for validation [27]. This first step was to confirm that the example of the base patient resource instance is FHIR-conformant. From the stakeholder interviews, we aggregated information data sets. We then mapped them to the standard patient resource to create a referral resource with extensions that capture all the identified data points. We further created the bare XML schema for easy file-based resource instance validation. The codes in XML and JSON formats are freely available on the GitHub directory [28]. Our steps and tools used are shown in Figure 1.

**Figure 2:**
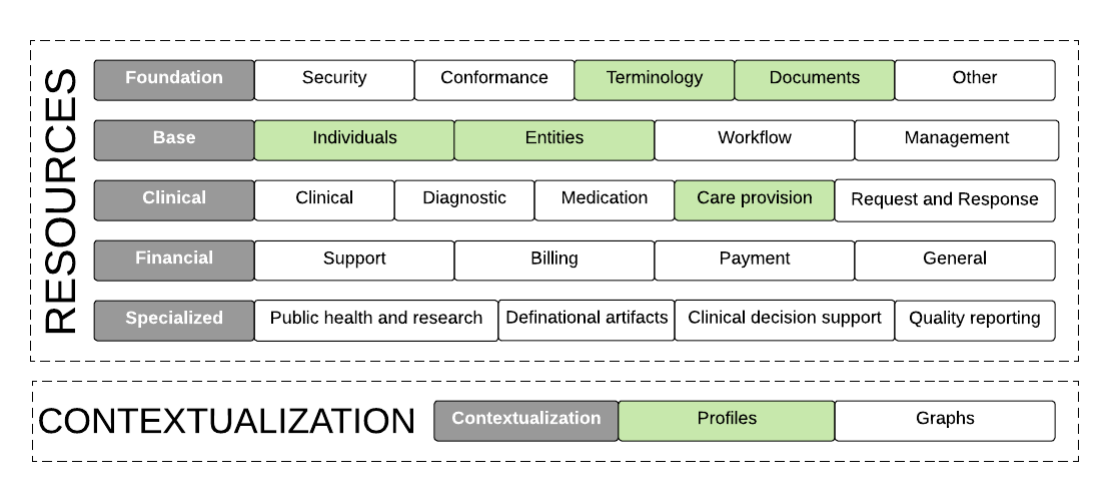
Resources considered for the Referral bundle.

We modeled the FHIR referral use case profile of information flow regarding pregnant women from one PHC center—for example, PHC center 1 to PHC center 2—or general hospital. Afterward, these resource mapping outputs were then synthesized into JSON and XML machine-readable data formats on the basis of FHIR resources for antenatal referral. We have further indicated the resources category affected by our referral bundle in green in Figure 2.

### Binding With ICD-10 Terminology

An example referral use case is described in Sierra Leone’s digital health strategy 2018-2023, “Table 2- Scenario: The vision in Practice” [12]. Asuma, a pregnant woman described in the use case, was referred to the clinic from the community by a roaming community health extension worker. See page 25 of Sierra Leone’s national digital health strategy for more information on this use case. In ICD-10, the “Personal History of malaria” code is Z8613. The code allows for unique identification in any information system using the same coding system, thus distinguishing this from, say, B500, which represents “Plasmodium *falciparum* malaria with cerebral complication,” which is a case of complicated malaria with intermittent coma. We analyzed the 21 chapters of ICD-10. Table 2 highlights those that are most relevant for in-depth study when designing a Maternal and Child Health (MNCH) information management system [10].

Code O98.6 represents “Protozoal disease complicating pregnancy, childbirth, and the puerperium.” This is synonymous with “Malaria in pregnancy” or “Maternal malaria during pregnancy,” both not explicitly coded in ICD-10 [29]. “Malaria in pregnancy” is the scenario described in the Sierra Leone MNCH digital health use case from the preceding paragraph.

**Table 2:** Key Maternal and Child Health chapters of the International Classification of Diseases, Tenth Revision coding system.

Chapter	Description	Code range
XV	Pregnancy, childbirth, and the puerperium	O00xx to O99xx
XVI	Certain conditions originating in the perinatal period	P00xx to P96xx
XVIII	Symptoms, signs, and abnormal clinical and laboratory findings not elsewhere classified	R00xx to R99xx
XX	External causes of morbidity and mortality	V01xx to Y98xx
XXI	Factors influencing health status and contact with health services	Z00xx to Z99xx

### Ethics Consideration

This study was exempted from ethics approval by the University of Malta ethics review board.

## Results

### Survey Outputs

In addition to responses from these surveys, 3 different referral forms for referral tracking among pregnant women used in the state were made available by respondents. The forms were then mapped to survey questions to generate a unified form with a union of content from the 3 forms in Table 3.

Survey responses from care providers varied widely and included extraneous information than in the referral forms. Based on the 24 health care providers' responses for the first 2 questions—“What Information is shared when referring a pregnant woman?” and “what information is expected when receiving a pregnant woman?”—none of the responses matched for all respondents. In response to question 3—“what forms are used?”—3 respondents said “referral letter” and 7 said “referral form.” Three respondents noted that referral forms varied by health institutions, while one indicated that they do not use any forms for referral. Other forms listed by respondents are the consent form, investigation form, chemistry form, hematology form, results form, ultrasound form, laboratory form, radiology form, and virology form. In response to the question, “What information is the client, or their caregiver expected to have or know?” At the same time, 2 respondents said “nill,” the rest of them listed information that completely varied. When we mapped the aggregated responses with the form details from Table 3, the list of data sets will be similar to that shown in Textbox 1.

**Table 3:** Form contents and their mapping.

Ebonyi State Ministry of Health referral slip	National Health Insurance Scheme (NHIS) referral form	Women, infants, and children referral form for pregnant women
Facility name	Facility name NHIS code	N/A^a^
N/A	Date	Measurement date
Patient number and social insurance number	Health Management Organization (HMO) NHIS ID number HMO code	—
Name of patient	Name	Patient's name (last, first)
Age	Date of birth	Date of birth (MM/DD/YY)
Sex	Sex	—
Address	N/A	Address (state, city, zip code)
N/A	N/A	Telephone number
Complaints	Presenting complaint	—
Findings on examination	Examination findings	Height, weight Hemoglobin (g/dL), hematocrit (%), and blood test date
Investigations performed, if any	Investigation results	—
Provisional diagnosis	Provisional diagnosis	—
N/A	Reason for referral post medical history taking Clinical warnings (allergies)	Estimated date of confinement Date when last pregnancy ended Gravida Para Pregravida weight (lbs) Indicate any of the following medical conditions (diabetes, multiple pregnancies, hypertension, tuberculosis, previous poor pregnancy outcome, and history (specify) If other, current history of the condition (specify)
Current and recent medication	N/A	Current medication and supplements prescribed
N/A	Other relevant information	Impressions and comments
Name of officer	Referring doctor Medical and Dental Council of Nigeria number Receiving doctor’s Medical and Dental Council of Nigeria number Date	Name of the physician care provider group and clinic
Designation	N/A	—
Signature	Signature and stamp	Health care provider Signature Date
To	Health facility NHIS code	—

^a^N/A: not applicable.

**List of data sets.**

- Source health facility name
- Source health facility ID
- Destination health facility name
- Destination health facility ID
- Date of referral
- Patient name
- Patient number
- Patient no type (Health Management Organization, National Health Insurance Scheme, softwareVendor)
- Patient age
- Sex
- Address
- Complaint
- Presenting complaint
- Investigation done
- Findings on examination
- Provisional diagnosis
- Reason for referral
- Current medication
- Recent medication
- Name of referring officer
- ID of referring officer (Medical and Dental Council of Nigeria)
- Designation of the referring officer
- Other relevant information
- Referrals direction (in or out)
- Referrals by disease
- Malaria case referred for adverse drug reaction (Health Management Information System)
- Referral disease (tracked by age and case)

### Referral FHIR Resource (Known as Referral Letter or Discharge Letter)

**Figure 3:**
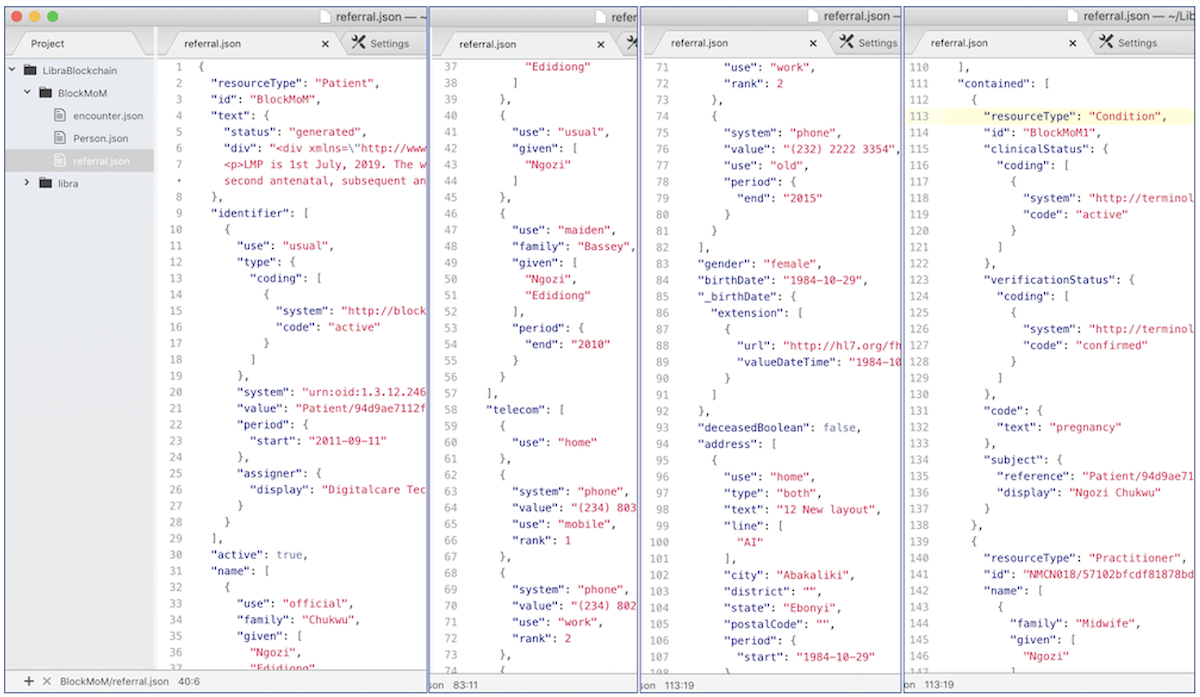
The profiled referral resource in picture.

The profile developed in this paper is only considered a provisional national profile suggestion for consideration and should not be relied on for clinical decisions. The resource mapping's final output is in XML and JSON formats and is freely available on GitHub [28]. The resource file is shown in Figure 3.

### REST and FHIR

Our artifact assumes the use of the REST paradigm for information exchange. In this section, we explain the technicalities of REST. REST is the foundation for the scale of the internet as we know it today [30]. While FHIR supports many different communication paradigms, REST is responsible for its popularity. The REST paradigm leverages the HTTP protocol with the simple client-server architecture with variants of catch-less/catching, stateless/stateful, or n-tired architectures and hierarchies. For further technical details of these ICT concepts, please refer to Thomas Fielding’s thesis introducing REST in 2000 [30].

Similar to traditional REST, FHIR’s REST paradigm considers entities and concepts as resources. Each of the resource instances is unique and is represented using a uniform resource identifier (URI). The URI may also be used for locating the resource if it points to the location on a given server (in which case, it can also be referred to as URL). There are a finite number of ways a client can manipulate entities and concepts (resources) located in an FHIR server using REST requests of get, put, post, delete, options, head, trace, and connect.

**Figure 4:**
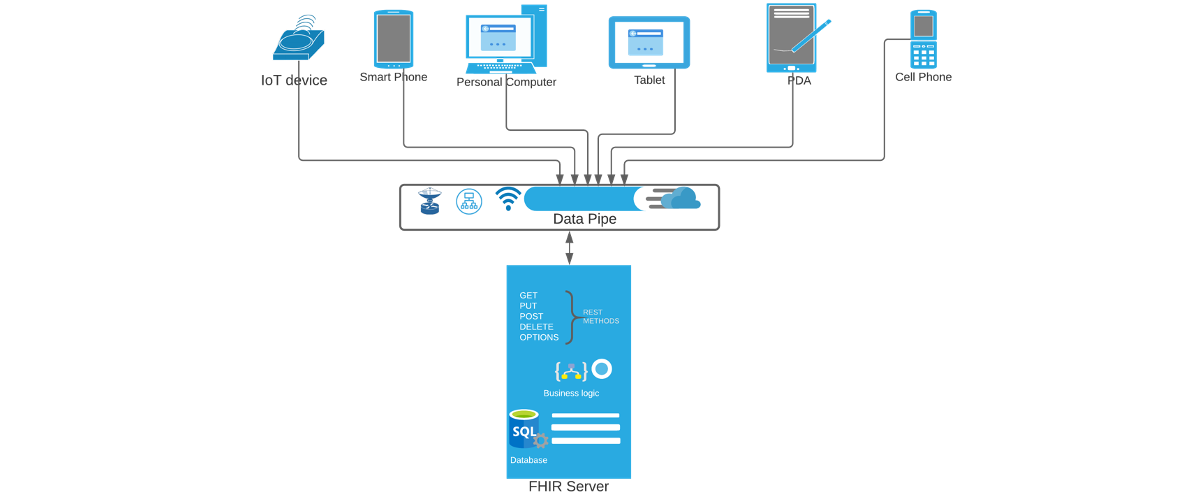
A typical client-server REpresentational State Transfer interface. FHIR: Fast Healthcare Interoperability Resource; IoT: Internet of Things; PDA: personal digital assistant.

The server responds to the client’s HTTP requests after performing internal business logics unique to each server implementation. The clients will receive the same response for similar HTTP requests irrespective of whether they are for a mobile app, web browser, computer application, or embedded device, as shown in Figure 4.

Both the client and server use the header and body components of the HTTP request or response for their information exchange, depending on the HTTP method used. The server always returns a status code indicating success or failure or a variant of either with further detail for each request. FHIR servers can use the OperationOutcome resource to provide structured details of request failure to the clients in the event of failure. There are over 100 different FHIR resources [31]. When the request succeeds, the client is sent the resource by the server.

Methods may or may not be allowed (or even implemented) by the server for a particular resource and may be specified by the client's server response. The header has many attributes that can be set, for instance, to indicate the data type it accepts, authorization credentials, connection, content encoding, caching, and more. The content-type attribute can indicate the resource as either XML or JSON format—both native to FHIR. FHIR, similar to REST, is an open standard and thus aligns with key principles of digital development [32].

## Discussion

### Principal Findings

We could not use the core FHIR resource because it did not contain all the data sets as aggregated in Figure 4. To add and extend the outstanding data sets, FHIR designers provide for and allow extendibility using profile extensions. FHIR contributors and balloting-process use the 80-20 rule to determine what makes it into the core FHIR resource [33]. It is understandable if there are no contributions from Nigeria or many other LMICs because they are often not represented at HL7 FHIR balloting. Our referral form reviews and data set mapping also lay credence to our hypothesis that the core patient resource needs an extension for our use case.

Our study shows that traditional paper referral forms currently in use vary widely in Nigeria’s PHC system as illustrated in the word art in Multimedia Appendix 1. The implication is that interinstitution care coordination will remain suboptimal as much of the essential information will be missing. This work will help policy makers and PHC centers in Nigeria understand the need to standardize or enforce agreed referral standards. In addition, the steps we have outlined in this work will help guide institutions as they standardize or adopt FHIR.

While the surveys were conducted before the COVID-19 pandemic, their findings are relevant for continued functioning of the PHC centers even amid and after the COVID-19 pandemic. The pandemic has exposed the weakness of health systems and shown the importance of interconnected and interoperable health systems. Emerging technologies are being proposed in response to the pandemic [34] and new models are emerging for health information interoperability [35] in LMICs. Even when PHC centers are digitized, referrals among health facilities in many LMICs with different software vendors do not happen seamlessly. Our research shows that referral practices for pregnant women varied significantly even in urban settings.

The key output of this study is the FHIR referral resource artifact, which will help vendors design consistent referral data sets and ensure out-of-the-box interoperability. This resource remains broad and from the core patient resource. FHIR allows for organizational or national extensions and adaptations [33]. Health authorities in many LMIC countries will benefit from standardizing and exposing its required referral data model for Women and Child Health, which encompasses MNCH [24]. Publishing a public FHIR specification that can be leveraged by MNCH solution implementers will help simplify interface implementations.

**Figure 5:**
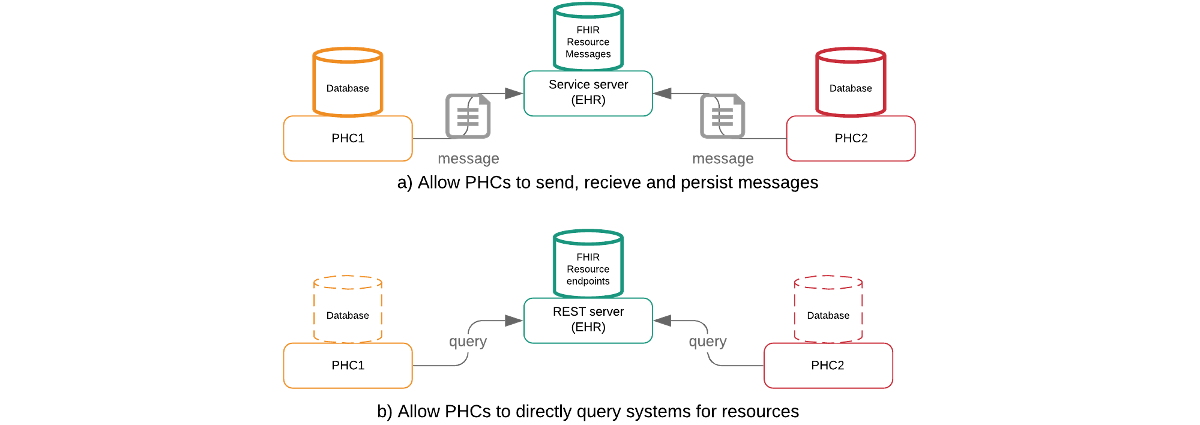
Fast Healthcare Interoperability Resource (FHIR) paradigms in a 2–public health care (PHC) system referral exchange scenario. EHR: electronic health record. REST: REpresentational State Transfer.

We here illustrate that our approach differs from traditional health information exchange approaches. Figure 5A shows the traditional document- and message-based information (which is still supported by FHIR) where both databases are required to retain the messages being exchanged. Figure 5B shows that the end point query approach using REST method calls is used to access or share FHIR resources being exchanged.

Women continue to die owing to preventable causes at the point of giving birth. Many of these deaths happen before, during, and after delivery. In addition, maternal health has been highlighted in some LMIC national digital health strategies (ie, Nigeria and Sierra Leone) as a priority health area [11]. Furthermore, our BlockMom model used the ICD terminology over the SNOMED-CT model owing to its favorable pricing license. For instance, SNOMED licenses are based on the number of health facilities using the terminology service, though there are requirements to apply for a waiver for certain implementations in LMICs. Moreover, the model’s deliberate focus on FHIR over other HL7 or ISO standards was because it is free and open for adaptation, adoption, and testing in LMICs.

### Limitations

We used on-file schema and the ICD-10 version 2021 text file to validate profiled resources. Even though the bare schema was provided in the GitHub directory, standard practice would be to set up an FHIR server for this purpose. This is the focus of our future study. Although many mobile-based solutions are available in PHC centers in LMICs, these profiles can only be used with a mobile-based solution that uses patient-specific information rather than aggregated information, as is the case with many community health worker information systems. Furthermore, ICD-11 2022 release has just been launched by the WHO, which emphasizes following the profiling process and not necessarily the output [36].

We also noted that unique identification metrics and characteristics, as explained by McFarlane et al [37] and Chukwu [38], were not part of the data sets proposed and made available during referral. This aspect was profiled as part of the FHIR resource. Besides, this may be the case as the patient-required abridged historical information is assumed to be comprehensive enough. Nevertheless, in a digital platform where a unique identifier is important, this assumption will not hold true. We assumed cryptographically generated unique identification mechanisms in this prototype.

A key limitation of our survey approach was that we did not prevalidate the questionnaire before use. In addition, our sampling methodology used a snowball strategy that targeted health workers from the most urban part of Ebonyi State, Nigeria. We are aware and acknowledge that this may seem inherently biased. However, our aim for the survey was to determine consistency or otherwise of the referral data sets, which we determine to vary widely across all respondents. In addition, since Abakaliki is the state capital and the main metropolitan city in the state, it is expected to have a standardized referral form; however, it does not.

### Conclusions

Questionnaire responses were collected from health care providers, and referral forms from health institutions in Nigeria were reviewed. Survey responses and fields of referral forms show variability in referral data sets across respondents and forms. Here we have made a case for FHIR, an emerging health care data interchange standard, and have profiled a referral resource for PHC information exchange targeted at LMICs. This paper describes the profiling steps, including key questionnaire responses and mapping of referral forms. We have proposed the use of ICD-10 terminology and used file-based schema validation. The methodology and artifacts will be invaluable for the research and implementation community targeting LMICs. Our future work will set up the server and configure the appropriate binding for this and other resources.

## Appendix

Multimedia Appendix 1 Survey respondents' referral keywords.

## Abbreviations

CEN: European Committee for Standards’ Health ICT
DICOM: Digital Imaging and Communication in Medicine
EHR: electronic health record
FHIR: Fast Healthcare Interoperability Resource
ICD: International Classification of Diseases
ICT: Information and Communication Technologies
ISO: International Organization for Standards
LMIC: low- and middle-income country
LOINC: Logical Observation Identifiers Names and Codes
MNCH: Maternal and Child Health
PHC: primary health care
REST: REpresentational State Transfer
RHIS: routine health information systems
SNOMED-CT: Systematized Nomenclature of Medicine–Clinical Terms
URI: uniform resource identifier
WHO: World Health Organization
