# Supplementary figures and images for: Standardizing Primary Health Care Referral Data Sets in Nigeria: Practitioners' Survey, Form Reviews, and Profiling of Fast Healthcare Interoperability Resources (FHIR)
Source: JMIR Form Res. 2022 Jul 7;6(7):e28510. doi: 10.2196/28510 (PMC9305397; doi:10.2196/28510)

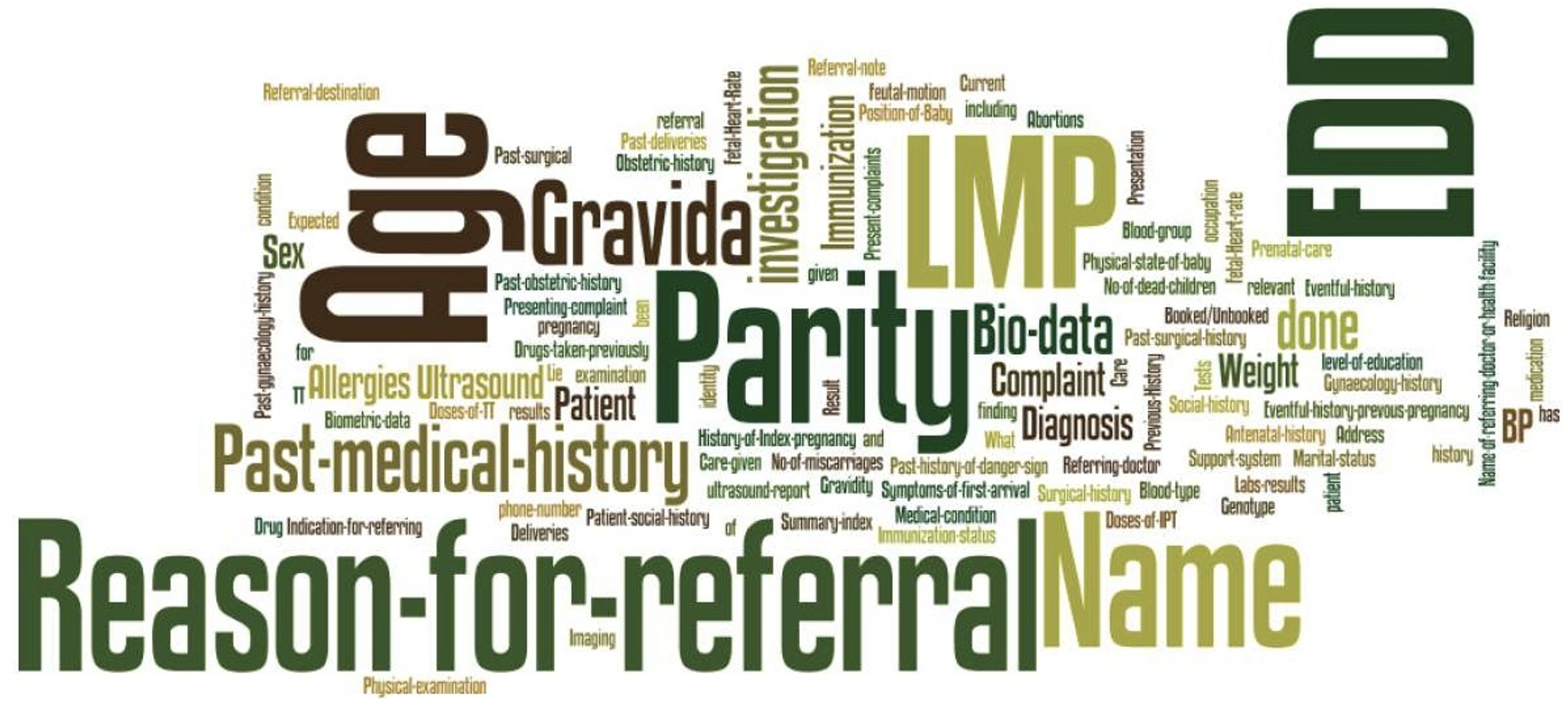

Supplement: Multimedia Appendix 1 [file formative_v6i7e28510_app1.png]
